# Overview of the human pharmacokinetics of recombinant activated factor VII

**DOI:** 10.1111/j.1365-2125.2007.03030.x

**Published:** 2007-10-24

**Authors:** Thomas Klitgaard, Tina G Nielsen

**Affiliations:** Novo Nordisk A/S Bagsværd, Denmark

**Keywords:** bleeding rate, NovoSeven®, pharmacokinetics, plasma clearance rate, recombinant activated factor VIIa

## Abstract

**AIMS:**

To review the pharmacokinetics of rFVIIa in various patient populations, and to discuss the differences observed between groups.

**METHODS:**

Based on a registry of Novo Nordisk studies, 14 studies evaluating rFVIIa pharmacokinetics following single and multiple bolus administration in healthy volunteers, adult and paediatric patients with congenital haemophilia and inhibitors, patients undergoing liver surgery and in patients with cirrhosis, inherited FVII deficiency, upper gastrointestinal bleeding or severe trauma were identified. Data on rFVIIa PK, analyzed with noncompartmental and population pharmacokinetic methods, were extracted.

**RESULTS:**

Plasma clearance was a more robust parameter than half-life for comparing rFVIIa pharmacokinetics between groups. In healthy volunteers and patients with no or low-level bleeding (e.g. adults with haemophilia, nonbleeding patients with cirrhosis), plasma clearance was relatively low (30–40 ml kg^−1^ h^−1^). In children with haemophilia and adults with high-level bleeding (e.g. cirrhotic patients undergoing orthotopic liver transplantation or resection) and patients with congenital FVII deficiency, plasma clearance was relatively higher (60–90 ml kg^−1^ h^−1^).

**CONCLUSIONS:**

Comparison of plasma clearance rates in different patient populations suggested that subjects fall into two distinct groups. These differences may have clinical implications in terms of how to adapt the rFVIIa dosing regimen, depending on the expected bleeding rate/blood loss and underlying disease.

## Introduction

Recombinant activated factor VII (rFVIIa, NovoSeven®; Novo Nordisk A/S, Bagsværd, Denmark) is established for the treatment of bleeding episodes and for the prevention of bleeding during surgery or invasive procedures in patients with congenital haemophilia A and B with inhibitors to coagulation factors VIII (FVIII) or IX (FIX) or in those expected to have a high anamnestic response to FVIII or FIX, acquired haemophilia, congenital FVII deficiency or Glanzmann's thrombasthenia refractory to platelet transfusions.

In addition to these indications, the therapeutic potential of rFVIIa has been explored for a number of critical bleeding situations. For example, rFVIIa successfully reversed coagulopathy in 75% (61/81) of patients with bleeding and coagulopathy resulting from a number of different causes, including acute traumatic haemorrhage and traumatic brain injury [1]. In a multicentre, random ized, controlled Phase II trial, rFVIIa reduced red blood cell (RBC) transfusion requirements and the need for massive transfusion *vs.* placebo in patients with severe blunt trauma [2] and a confirmatory Phase III trial evaluating rFVIIa for the treatment of traumatic haemorrhage is ongoing. In addition rFVIIa has been shown to reduce haematoma growth in intracerebral haemorrhage, although this reduction did not translate into improved long-term clinical outcome [3]. A number of clinical guidelines on rFVIIa use in nonhaemophilia indications has been published [4, 5].

At pharmacological doses, rFVIIa binds to the surface of locally activated platelets following vascular injury, directly activating factor X, and thereby enhancing localized thrombin generation and formation of a stable fibrin clot only at the site of vascular injury [6]. Although not the focus of this paper, pharmacokinetics are relevant for an understanding of drug safety and the incidence of drug related adverse events [7]. In addition, by improving the understanding of drug distribution and elimination processes of rFVIIa, pharmacokinetics may offer insight into the design of optimal therapeutic regimens for this unique agent, particularly in cases of severe or poorly characterized bleeding. This paper aims to address this by reviewing pharmacokinetic data from clinical studies of rFVIIa following single and multiple i.v. bolus administration. The findings and their relevance to the therapeutic use of rFVIIa are discussed.

## Overview of studies evaluating the pharmacokinetics of rFVIIa

The pharmacokinetics of rFVIIa have been assessed in studies of healthy volunteers and in studies of patients with no or only minor tissue injuries and low associated levels of bleeding, as well as in patients with more pronounced tissue injuries and bleeding, a distinction that appears to have implications for the pharmacokinetics of rFVIIa. Patients received either a single i.v. bolus [8–17] or multiple i.v. boluses of rFVIIa [2, 18–20] (details of individual studies are shown in Table 1). Pharmacokinetic studies were identified based on a registry of Novo Nordisk files.

**Table 1:** Overview of studies evaluating the pharmacokinetics of rFVIIa

Study description	*n*	rFVIIa dose, μg kg ^−1^*	Timing of sample collection	PK analysis	CL (ml kg^−1^ h^−1^)†	*V*_ss_(ml kg^−1^)	*t*_1/2_(h) (*t*_1/2β_[h])‡
**Healthy Japanese and Caucasian volunteers [16]**	29	40, 80, 160	0, 10, 30 min, 1, 2, 3, 5, 8, 12, 24 h	NCA	34–37	130–165	3.90–5.99 `
**Healthy volunteers [11, 12]**	28	5–20	0, 5–10 min, 2, 3, 5, 6, 8, 12 h	PopPK	31–35	80.0 (≤20 μg kg^−1^)	2.43 (≤20 μg kg^−1^)‡
		20–320				93.6 (>20 μg kg^−1^)	2.45 (>20 μg kg^−1^)‡
**Comparison study – Adults and children with haemophilia A [15] (Kristensen, Klitgaard, data on file 2004)**	5 (Adults) 12 (Children)	90 (Adults) Two doses, 90 and 180 (Children)	0, 10, 20 min, 1, 3, 6, 8, 12 h	NCA	58 (Children, FVII:C)	164 (Children, FVII:C)	2.6 (Children, FVII:C)
					39 (Adults FVII:C)	128 (Adults FVII:C)	3.1 (Adults FVII:C)
					78 (Children, FVIIa clot)	196 (Children, FVIIa clot)	2.3 (Children, FVIIa clot)
					53 (Adults, FVIIa clot)	159 (Adults, FVIIa clot)	2.3 (Adults, FVIIa clot)
				PopPK	60 (Children, FVII:C)	182 (FVII:C)	4.3 (Children FVII:C)‡
					44 (Adults FVII:C)	215 (FVIIa clot)	4.8 (Adults FVII:C)‡
					84 (Children, FVIIa clot)		2.5 (Children FVIIa clot)‡
					60 (Adults, FVIIa clot)		3.1 (Adults FVIIa clot)‡
**Adults with haemophilia A or B [9] (Jansen, data on file 2001)**	15	17.5, 35 or 70	0, 10, 20, 50 min, 2, 4, 6, 8, 12, 24 h	NCA	32–37	103.5–110.0	2.48–2.82
	14			PopPK	29 (bleeding)	174.5§	4.37 (bleeding)‡
					33 (nonbleeding)		5.34 (nonbleeding)‡
**Japanese patients with haemophilia A or B [14]**	8	120	0, 10, 15, 20 min, 1, 2, 4, 6, 8, 24 h	NCA	nc	nc	2.5–5.0
**Paediatric patients with haemophilia A or B [8]**	6	114–196	0, 5, 10, 30 min, 1, 2, 3, 4 h	NCA	67	nc	1.32
**Patients with inherited FVII deficiency [13]**	5	15, 30	0, 10, 15, 30 min, 1, 2, 4, 6, 8, 10, 24 h	NCA	71 (15 μg kg^−1^, FVII:C)	280 (15 μg kg^−1^, FVII:C)	2.82 (15 μg kg^−1^, FVII:C)
					65 (15 μg kg^−1^, FVIIa clot)	210 (15 μg kg^−1^, FVIIa clot)	2.49 (15 μg kg^−1^, FVIIa clot)
					79 (30 μg kg^−1^, FVII:C)	290 (30 μg kg^−1^, FVII:C)	3.11 (30 μg kg^−1^, FVII:C)
					68 (30 μg kg^−1^, FVIIa clot)	230 (30 μg kg^−1^, FVIIa clot)	2.62 (30 μg kg^−1^, FVIIa clot)
**Nonbleeding patients with cirrhosis and prolonged PT [10]**	10	5, 20, 80	0, 10, 30 min, 2, 4, 6, 8, 12 h	NCA	33–44	102–175	2.37–3.23
**Patients with cirrhosis and UGIB [19/Unpublished data] (Klitgaard, data on file 2004)**	234	100 multiple doses	0, 4, 12, 24 h, days 2, 3, 4, 5 subgroup additional samples: 10, 30 min, 1, 2, 30 h	PopPK	78	344	3.08¶
**Patients with cirrhosis and UGIB [18]**	10	80	0, 0.5, 2, 4, 6, 8, 12 h	NCA	nc	nc	Approx. 2–3
**Patients undergoing OLT – single dose study [17] (Jansen, data on file 2001)**	55	20, 40 or 80	0, 15, 60 min, then hourly till end of surgery, 24 h, days 3, 7	PopPK	62 (−liver)	152.2	2.59 (−liver)‡
					90 (+liver)		2.19 (+liver)‡
**Patients undergoing OLT – multiple dose study [20] (Erichsen, Klitgaard, data on file 2004).**	112	60 or 120 multiple doses	0, 15, 60 min, then hourly till end of surgery, 24 h, days 3, 7	PopPK	75 (RBC = 0) increases with RBC decreases with body weight	154	1.43‡
**Noncirrhotic patients undergoing liver resection [27] (Klitgaard, data on file 2005)**	125	20, 80 multiple doses	0, 15, 60 min, then hourly till end of surgery (last sample 5 h NCA, 6 h PopPK)	NCA	64 (20 μg kg^−1^)	124 (20 μg kg^−1^)	1.08 (20 μg kg^−1^)
					64 (80 μg kg^−1^)	277 (80 μg kg^−1^)	1.57 (80 μg kg^−1^)
				PopPK	70	121.5 (20 μg kg^−1^)	1.20 (20 μg kg^−1^)¶
						164 (80 μg kg^−1^)	1.62 (80 μg kg^−1^)¶
**Patients with traumatic blunt and/or penetrating injury [2, 23]**	21	3 doses (200 at 0 h; 100 at 1 and 3 h)	0, 30 min, 1, 2, 3, 4, 6, 8, 12 h (frequent sampling)**	NCA	41 (blunt)	nc	nc
					40 (penetrating)		
	230			PopPK	40 (RBC = 8.7 units) increases with RBC	120	2.37

* Single dose unless stated otherwise.

† FVII:C assay unless stated otherwise.

‡ *t*_1/2β_[h] calculated, rather than terminal *t*_1/2,z_ (h).

§ Calculated post hoc as the sum of the compartment volumes.

¶ Calculated post hoc from the reported estimates of distribution volume and clearance.

** 21 of the 43 subjects in the frequent-sampling group were dosed with rFVIIa and these data were analyzed noncompartmentally (n = 21). Data from both frequently sampled and nonfrequently sampled subjects were analyzed in PopPK (total n = 230). CL, plasma clearance; rFVIIa, recombinant activated factor VII; FVII:C, FVII coagulant activity; nc, not calculated; NCA, Noncompartmental analysis; OLT, orthotopic liver transplantation; PK, pharmacokinetic; PopPK, Population pharmacokinetic (analysis); RBC, red blood cell(s); *t*_1/2_, plasma half-life, GIB, upper gastrointestinal bleeding; V_ss_, steady state volume.

In all studies, pharmacokinetic assessments were based on the FVII coagulant activity (FVII:C) assay, a one-stage assay using thromboplastin tissue factor, which quantifies FVII clotting activity in plasma (Capio Diagnostik A/S, Denmark) [21]. The lower limit of quantification for the assay is 0.06 U ml^−1^, and the assay precision is 10% CV. Since this assay does not distinguish endogenous FVII/FVIIa from rFVIIa, baseline plasma FVII:C (i.e. before administration of rFVIIa) was taken into account in the pharmacokinetic analyses. Some studies also used the FVIIa clot activity assay, which assesses FVIIa exclusively [21], and has a lower limit of quantification of 0.02 IU ml^−1^ and an assay precision of 6% CV. The FVIIa clot activity assay is more accurate than the FVII:C assay, particularly at FVII concentrations below 6 IU ml^−1^[15, 22].

Results were generated using noncompartmental analysis (NCA) and/or population pharmacokinetic analysis of compartmental models (PopPK) (Table 1). The NCA method requires rich concentration–time profiles for each individual and is therefore not applicable where data are limited. PopPK analysis does not have this constraint, allowing even sparse profiles to be analyzed. All population models used in the PopPK analyses of rFVIIa had first-order elimination from the central compartment and baseline to account for endogenous FVIIa production, if relevant. A two-compartment population model was used for healthy volunteers [12], one study of patients undergoing orthotopic liver transplantation (OLT) [17], patients with severe trauma [23] and patients with haemophilia. A one-compartment model was used for one study of patients undergoing OLT [20], patients with upper gastrointestinal bleeding (UGIB) and patients undergoing liver resection.

## rFVIIa in healthy volunteers

Several studies have examined the pharmacokinetics of rFVIIa in healthy adult volunteers. The most recent showed that healthy Japanese and Caucasian volunteers had comparable estimates of plasma clearance (33–37 ml kg^−1^ h^−1^), terminal half-life (3.90–05.99 h^−1^) and steady-state volume of distribution (130–165 ml kg^−1^). No sex-related differences in these parameters were observed [16]. These results are consistent with those in healthy Caucasian volunteers who were anticoagulated with acenocoumarol to international normalized ratio 2–2.5 prior to dosing [11]. Analysis using a two-compartment model provided estimates of half-life, clearance and steady-state volume of distribution for doses above and below 20 μg kg^−1^[12]. Clearance rates of 31–35 ml kg^−1^ h^−1^ were reported, which were comparable with those observed in the study by Fridberg *et al.*[16].

## rFVIIa in patients with minor tissue injury and low-level bleeding

### Adults with haemophilia A or B

Analysis of rFVIIa pharmacokinetics in adults with haemophilia has demonstrated clearance rates of 32–39 ml kg^−1^ h^−1^, according to NCA [9, 15], and 29–44 ml kg^−1^ h^−1^ according to PopPK (Jansen, data on file 2001; Kristensen, Klitgaard, data on file 2004).Pharmacokinetic data from Japanese adult patients with severe haemophilia A or B and inhibitors were consistent with those for Caucasian subjects [14], with a plasma half-life of 2.5–5.0 h (mean 3.5 h) by NCA.

### Children with haemophilia A or B

Analysis of rFVIIa pharmacokinetics in children with haemophilia A [15] or haemophilia A or B [8] provided estimated clearances of 58 and 67 ml kg^−1^ h^−1^ (NCA; FVII:C), respectively. A similar clearance rate (60 ml kg^−1^ h^−1^) was reported by PopPK analysis of data from Villar *et al.*[15] (Kristensen, Klitgaard, data on file 2004).

**Figure 1:**
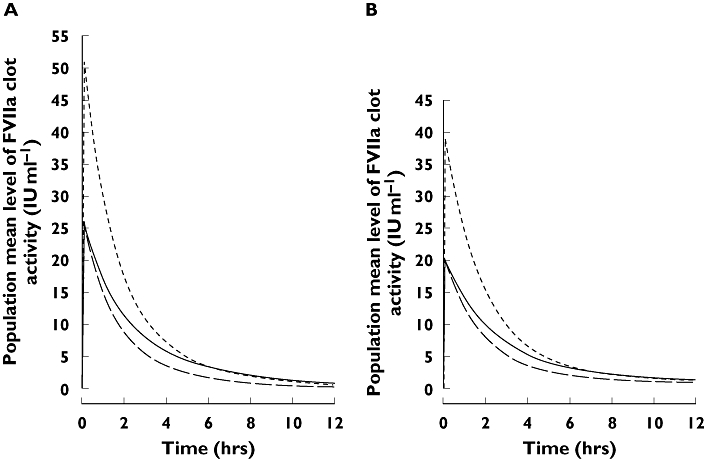
Population pharmacokinetic profiles of rFVIIa for patients with haemophilia A *vs.* time, following 90 μg kg^−1^ (adults and children) and 180 μg kg^−1^ (children only) rFVIIa. Based on PopPK analysis of (A) FVIIa clot activity and (B) FVII:C activity in Villar *et al.*[15]. Adults 90 μg kg^−1^, (—); Children 90 μg kg^−1^, (––); Children 180 μg kg^−1^, (- - - -)

**Figure 2:**
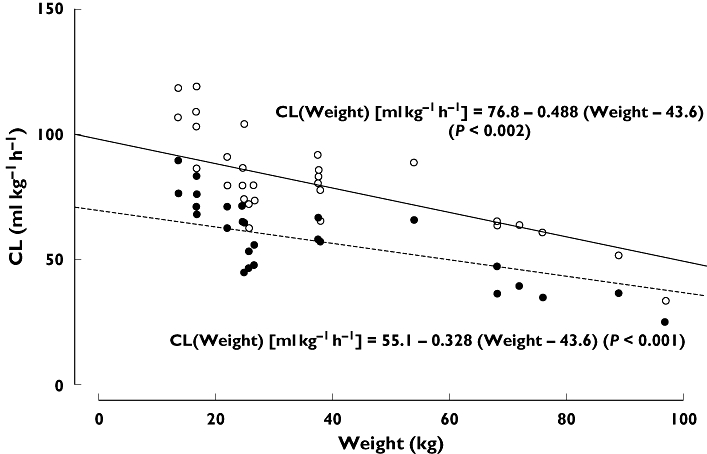
Individual estimates of clearance (CL) *vs.* weight for adult and paediatric patients with haemophilia, based on PopPK analysis of data from Villar *et al.*[15]. Estimated linear relationship between CL and weight based on FVIIa clot activity assay (full line, open circles) and FVII:C activity assay (dashed line, filled circles). Based on FVIIa, (○—); Based on FVII:C, (• - - - -)

Direct comparison of the results for adults and children (aged 18–55 and 2–12 years, respectively) by Villar *et al.*[15] indicated that plasma clearance was significantly (*P* < 0.05; NCA) higher in paediatric patients than in adults, using both the FVII:C and FVIIa clot activity assays. In contrast, there was only a nonsignificant trend towards an increased volume of distribution in children compared with adults and no difference in terminal half-life between the two groups (both *P* > 0.05). Subsequent analysis of these results by PopPK revealed no statistically significant (*P* > 0.01) differences in volumes of distribution between adults and children for either assay, while plasma clearance rates per kg body weight were higher in children (Kristensen, Klitgaard, data on file 2004). This difference reflects a higher metabolic activity in children than in adults per kg body weight and is possibly related to age-related differences in body composition, including differences in liver volume per kg body weight, as previously described in the literature [24, 25]. Mean population pharmacokinetic profiles for children and adults are shown in Figure 1, and the relationship between clearance and weight is illustrated in Figure 2.

In Villar *et al.*[15], children received first 90 then 180 μg kg^−1^ (after washout). Analysis of the effect of dose demonstrated dose proportionality for the two rFVIIa doses evaluated. This is an important finding, as it indicates a predictable pharmacokinetic response in children to changes in dose.

### Patients with inherited FVII deficiency

In Berrettini *et al.*[13], rFVIIa 15 and 30 μg kg^−1^ was administered to each patient (aged 20–43 years) with a 3-day washout period between doses. No significant dose-dependent differences in clearance, half-life or volume of distribution were observed (*P* > 0.05). Plasma clearance rate was 71–79 ml kg^−1^ h^−1^ and hence higher than that observed in adult patients with haemophilia and in healthy subjects.

## rFVIIa in patients with cirrhosis

Patients with cirrhosis may have low concentrations of coagulation factors, including FVII [26], resulting in a prolonged prothrombin time (PT). In a preliminary dose escalation study, 10 patients with cirrhosis and prolonged PT with no bleeding [10], received three successive doses of rFVIIa (5, 20 and 80 μg kg^−1^) over 3 weeks. Plasma clearance rates ranged from 33 to 44 ml kg^−1^ h^−1^, corresponding to those for healthy adults and adults with haemophilia; half-life ranged from 2.37 to 3.23 h. The pharmacokinetic parameters assessed were independent of dose (*P* > 0.05).

### Patients with upper gastrointestinal bleeding

In contrast to nonbleeding patients with cirrhosis, the clearance rate was 78 ml kg^−1^ h^−1^ after eight doses of 100 μg kg^−1^ rFVIIa over a 30 h period in patients with cirrhosis and UGIB [19] (Klitgaard, data on file 2004). This is approximately double that reported in healthy adults, and was similar to clearance rates in patients with FVII deficiency (Klitgaard, data on file 2004). However, the half-life (3.08 h) was within the range reported for healthy adults. A preliminary open-label study in a similar patient population reported a slightly lower half-life of 2–3 h following administration of a single dose of 80 μg kg^−1^ rFVIIa [18].

### Patients undergoing orthotopic liver transplantation

**Figure 3:**
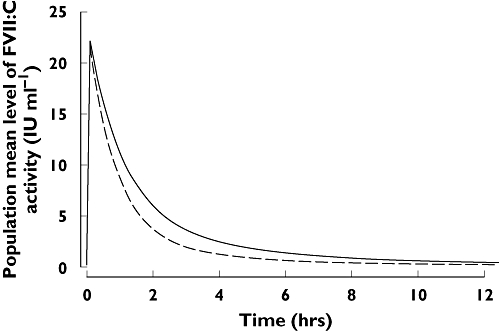
Population pharmacokinetic profiles of rFVIIa during phases with and without liver circulation, for patients undergoing orthotopic liver transplantation, following a single dose of 80 μg kg^−1^ rFVIIa. Analysis of data from Planinsic *et al.*[17]. (Erichsen, Klitgaard, data on file 2004) No liver circulation, (—); Liver circulation, (––)

Two exploratory studies have assessed the effect of rFVIIa in patients with cirrhosis undergoing OLT. In Planinsic *et al.*[17], shortly after patients received a single dose of rFVIIa before surgery, circulation through the liver was stopped, and the liver was removed and replaced with the donor liver, which was then reperfused. This enabled subsequent assessment of clearance with and without circulation through the liver. The results showed significantly (*P* < 0.005) different plasma clearance during periods of no circulation (62 ml kg^−1^ h^−1^) *vs.* when the liver was perfused (89 ml kg^−1^ h^−1^), while intercompartmental clearance was unaffected by the absence of circulation through the liver (*P* > 0.25) (Jansen, data on file 2001). Simulated population pharmacokinetic profiles corresponding to phases with and without liver circulation are illustrated in Figure 3. Clearance rates under both conditions were higher than in healthy volunteers and similar to those in patients with cirrhosis and UGIB.

In Lodge *et al.*[20], patients received multiple doses of rFVIIa immediately prior to first incision, then every 2 h until 30 min before anticipated liver reperfusion, with an additional rFVIIa dose at the end of surgery. In a subsequent analysis of the study data by PopPK, clearance was estimated to be 74 ml kg^−1^ h^−1^, in agreement with Planinsic *et al.*[17] (Erichsen, Klitgaard, data on file 2004). Analysis of the effect of a number of variables on plasma clearance revealed a significant effect (*P* < 0.01) of weight and RBC transfusion requirements. Plasma clearance increased by 4 ml kg^−1^ h^−1^ for each unit of RBC received, and decreased by 1.3% for each kg change in body weight below 75 kg.

## rFVIIa in noncirrhotic patients undergoing liver resection

In a further study by Lodge *et al.*[27], patients received 20 or 80 μg kg^−1^ rFVIIa by slow infusion 5 min prior to first skin incision, with a second dose 5 h later if the surgery was anticipated to last over 6 h. Clearance rate was approximately 64 ml kg^−1^ h^−1^ (NCA) and 70 ml kg^−1^ h^−1^ (PopPK) for both doses (Klitgaard, data on file 2004). However, volume of distribution increased significantly (*P* < 0.001) with dose. Analysis of the effect of various parameters on rFVIIa pharmacokinetics found no significant influence of RBC transfusion requirement, blood loss during surgery, weight, age or sex. Given that a two-compartment model could not be estimated in the noncirrhotic patients, possibly due to the relatively short sampling duration, it is conceivable that the one-compartment results are biased, generating a higher estimate of clearance and a lower estimate of half-life.

## rFVIIa in patients with severe trauma

Boffard *et al.*[2] and Klitgaard *et al.*[23] investigated rFVIIa in two parallel, multicentre, randomized, placebo-controlled studies in severe blunt and penetrating trauma. Patients who received six units of RBCs within a 4-h period were randomly assigned to receive either placebo or three doses of rFVIIa (200, 100 and 100 μg kg^−1^) at 0, 1 and 3 h, with the first dose administered immediately after transfusion of the eighth unit of RBC. Analysis by NCA of data for patients who underwent frequent blood sampling following rFVIIa dosing (*n* = 21) yielded clearance rates of 41 and 40 ml kg^−1^ h^−1^ for blunt and penetrating trauma, respectively. PopPK analysis of the larger data set of patients who underwent both frequent and less frequent blood sampling (*n* = 230) gave an estimated clearance rate of 40 ml kg^−1^ h^−1^.

Further analysis revealed a significant correlation between clearance and 48 h RBC transfusion (*P* < 0.001). Forty-eight h RBC requirement was used as a surrogate marker for bleeding rate, as this cannot be accurately estimated in trauma patients. It should, however, be noted that the 48 h RBC requirement may overestimate the actual rate of bleeding during the 12 h pharmacokinetic sampling period.

**Figure 4:**
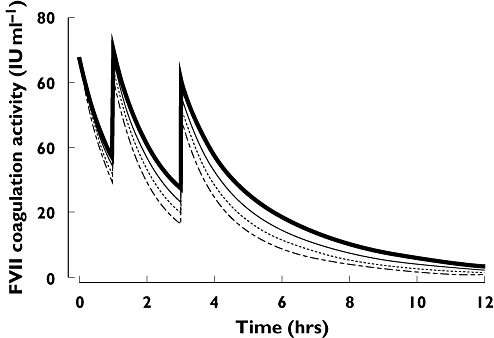
Population pharmacokinetic profiles of rFVIIa after administration of three doses (200, 100 and 100 μg/kg) at 0, 1 and 3 h, simulated for various postdose RBC transfusion requirements. Based on PopPK analysis of data in trauma patients [23]. Figure adapted from [23] by kind permission of BioMed Central. RBC = 8.7 units (mean), (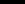); RBC = 20 units, (······); RBC = 30 units, (––); RBC = 40 units, (- - - -)

Using this model, a patient receiving no RBC transfusions or 40 units of RBC after the first dose of rFVIIa would have a predicted clearance of approximately 36 ml kg^−1^ h^−1^ and 62 ml kg^−1^ h^−1^, respectively. The effect on the simulated population pharmacokinetic profiles is illustrated in Figure 4. The high variation in clearance, and the implications for the pharmacokinetic profile, suggests that a repeat dose regimen may be required for maintaining a minimum effective FVII coagulant activity level throughout this heterogeneous patient population.

## Correlation between pharmacokinetic parameters and bleeding

Examination of plasma clearance values from studies of rFVIIa across a range of indications, from healthy volunteers to patients with severe trauma, suggests the existence of two distinct populations. In healthy volunteers, plasma clearance values were approximately 30–40 ml kg^−1^ h^−1^. Plasma clearance values for adult patients with haemophilia, who typically are classified as having no bleeding or only low-level bleeding, and for nonbleeding patients with cirrhosis are also within this ‘normal’ range. Clearance of rFVIIa appears to be unaffected by dose in the range of 20–320 μg kg^−1^.

In contrast, patients with liver disease and active, high levels of bleeding (e.g. cirrhotic patients undergoing OLT and noncirrhotic patients undergoing major liver resection) have much higher plasma clearance rates of between 60 and 90 ml kg^−1^ h^−1^. This is approximately twice that seen in patients with low-level bleeding or healthy volunteers. Moreover, in the OLT group, plasma clearance increased significantly after transplantation and during liver reperfusion, a finding which supports the hypothesis that the liver is the principal site of rFVIIa metabolism [28]. The finding of an association between increased RBC transfusion requirement and increased clearance in trauma patients provides further evidence to support the link between bleeding levels (as estimated from RBC transfusion requirements) and plasma clearance values.

The relationship between RBC requirements and clearance may well be multifactorial. In patients with massive bleeding, the combination of coagulation factor consumption due to continued clot formation, loss due to ongoing bleeding, and dilution due to fluid and blood component therapy may reduce the rFVIIa concentration to a potentially suboptimal level for effective clot formation [29].

Furthermore, underlying liver disease seen in some of these patients is likely to reduce the endogenous production of a number of key coagulation factors, including FVII, and may be associated with both thrombocytopenia and platelet dysfunction [30]. It could be hypothesized that reduced production of coagulation factors and functional platelets may lead to ineffective or reduced coagulation. However, the overall impact this may have on rFVIIa clearance in the liver-diseased patient remains speculative.

Three other patient groups also had increased clearance rates relative to normal levels, namely, patients with congenital FVII deficiency, paediatric patients with haemophilia, and patients with UGIB and cirrhosis.

In patients with congenital FVII deficiency, increased clearance rates of rFVIIa may be linked to the absence of endogenous production of functional FVII/FVIIa. In haemophilia A patients, following administration of rFVIIa 90 μg kg^−1^, significantly faster clearance was observed in children compared with adults, suggesting that higher doses of rFVIIa may be needed to achieve the same plasma concentrations as in adults [15]. Finally, in patients with cirrhosis and UGIB, clearance was increased, although bleeding and RBC transfusion requirements during the period of pharmacokinetic sampling were relatively small (average RBC requirement during first 24 h: 0.9 ± 1.8 units). At present it is not clear why the rFVIIa metabolism appears to be altered in these patients.

It appears from these studies that plasma clearance may provide more useful information than half-life for assessing the behaviour of rFVIIa following dosing. Half-life estimates are often only based on a few terminal timepoints, and short sampling profiles may bias half-life estimates downwards, as a pure elimination phase towards the end of the pharmacokinetic profile will not have been recorded. In addition, mean plasma half-life estimates may be affected by the modelling approach used to fit the available data. In contrast, plasma clearance is more robust as a parameter for comparing rFVIIa pharmacokinetics between patient groups.

In conclusion, this overview has demonstrated that human subjects may be stratified into two distinct groups in terms of rFVIIa pharmacokinetics. In one, comprising healthy volunteers, adult patients with haemophilia and nonbleeding patients with cirrhosis, plasma clearance is relatively low (30–40 ml kg^−1^ h^−1^). In the other, comprising children with haemophilia, patients with congenital FVII deficiency and patients with active, high levels of bleeding, plasma clearance appears to be higher (60–90 ml kg^−1^ h^−1^). Differences in rFVIIa pharmacokinetics among patient groups may have clinical implications in terms of how to adapt the dosing regimen, depending on the expected bleeding rate/blood loss and the underlying disease.
